# Prevalence, patterns, and perceived value of complementary and alternative medicine among HIV patients: a descriptive study

**DOI:** 10.1186/s12906-017-1928-4

**Published:** 2017-08-23

**Authors:** Mandreker Bahall

**Affiliations:** 1 School of Medicine and Arthur Lok Jack Graduate School of Business, University of the West Indies, St. Augustine, Trinidad and Tobago; 2 Department of Medicine, San Fernando General Hospital, Chancery Lane, San Fernando, Trinidad and Tobago; 3 House #57 LP 62, Calcutta Road Number 3, McBean, Couva, Trinidad Trinidad and Tobago

**Keywords:** Complementary and alternative medicine, Human immunodeficiency virus, Medicinal herb, Satisfaction, Side effects, Spiritual therapy

## Abstract

**Background:**

Use of complementary and alternative medicine (CAM) is widespread among different patient populations despite the availability of evidence-based conventional medicine and lack of supporting evidence for the claims of most CAM types. This study explored the prevalence, patterns, and perceived value of CAM among human immunodeficiency virus (HIV) patients.

**Methods:**

This quantitative descriptive study was conducted between November 1, 2014 and March 31, 2015 among a cross-sectional, convenience sample of attendees of the HIV clinic of a public tertiary health care institution. Face-to-face interviews using a 34-item questionnaire were conducted. Data analysis included descriptive statistics, chi-square tests, and binary logistic regression analysis.

**Results:**

CAM was used by 113 (32.8%) of a total of 343 HIV patients, but <1% informed their health care providers of CAM usage. Medicinal herbs were the most common type of CAM used (*n* = 110, 97.3%) followed by spiritual therapy (*n* = 56, 49.6%), including faith healing/prayer and meditation. The most used medicinal herbs were *Aloe vera* (*n* = 54, 49.1%), ginger (*n* = 33, 30.0%), and garlic (*n* = 23, 20.9%). The most used vitamins were complex B vitamins (*n* = 70, 61.9%), followed by vitamin A (*n* = 58, 51.3%), vitamin E (*n* = 51, 45.1%), and vitamin D (*n* = 42, 37.1%). Most CAM users continued using conventional medicine in addition to CAM and were willing to use CAM without supervision and without informing their health care provider. Patients were generally satisfied with CAM therapy (*n* = 91, 80.5%). The main reasons for CAM use were the desire to take control of their treatment (8.8%) or just trying anything that could help (18.8%). Main influences were the mass media (32.7%) and non-hospital health personnel (19.5%). Predictors of CAM use were being 30–50 years, married and having a secondary school education.

**Conclusion:**

About one-third of HIV patients used CAM, but virtually none informed their healthcare provider. Medicinal herbs were the most common type of CAM, followed by spiritual therapy and vitamins. A patient’s decision to use CAM was influenced for the most part by the mass media and non- hospital health care personnel.

## Background

Conventional medicine (CM) has improved patient outcomes (e.g. life expectancy, quality of life, and patient satisfaction) in various diseases. However, there remain many unresolved challenges, such as inadequacy, ineffectiveness, inaccessibility, side effects, and unfulfilled patient expectations [1, 2]. Many patients infected with human immunodeficiency virus (HIV), have turned to complementary and alternative medicine (CAM) [3, 4] because of its perceived claims, namely curing [5], counteracting the side effects of CM [6–8], providing and promoting wellness and holistic care [6, 9] and treating a number of irreversible and chronic conditions such as HIV infections [10]. CAM is defined as “a group of diverse medical and health care systems, practices, and products that are not generally considered part of conventional medicine” [11]. It comprises herbs, dietary supplements, meditation, biofeedback, hypnosis, acupuncture, Ayurveda, homeopathy, naturopathy, Chinese medicine, chiropractic, massage, *tai chi*, yoga, electromagnetic therapy, kinesiology, *reiki*, and *qigong*.

The global prevalence of CAM varies with cultures, countries, and the interpretation of CAM, ranging from 9.8% to 76.0% [12]. The prevalence of CAM has been reported to be 38% among adults in the United States [13], 51.8% in the United Kingdom [14], and 68.9% in Australia [15]. In Trinidad and Tobago, the prevalence of CAM is 30.4% among asthmatics (for herbal medicines) [16], 56% among cardiac patients (for any type of CAM) [17], 24% among diabetic patients attending chronic disease clinics (for herbs) [18]. On average, 60% of HIV-positive individuals use CAM to treat HIV-related health concerns in the United States [8, 19, 20]. However, no studies have explored CAM usage among HIV patients in Trinidad and Tobago.

Trinidad’s traditional medicine (nonconventional indigenous medical practices) [21] and home medication/remedies [22, 23] have been in use by Amerindians (e.g. Tainos and Kalinagos), Afro-Trinidadians, and Indo-Trinidadians for centuries, and are still used in part due to the lack of available conventional health care [24] and the influence of positive testimonies and perceived benefits. Hsu et al. [25] showed that some patients with back pain feel cured with CAM, while others feel stronger, happier, livelier, and more motivated to live. Some patients also report stress relief [6]. With CM, patients benefit from improved quality of life, decreased mortality, decreased morbidity, and increased life expectancy [26, 27]. Nonetheless, HIV patients have numerous concerns, including depression [28, 29], stress and other psychological problems [30, 31], neglect by family members [32, 33], and limited or no resources [34]. Much of patient’s social [35] and spiritual needs [36] are ignored by CM practitioners, thus creating major gaps in patient health care [37]. Customer centredness was identified as a major indicator of quality health care by the United States Institute of Medicine [38].

HIV prevalence in Trinidad is on the rise [39] even though disease complications are decreasing [26]. Improvement of clinical outcomes is largely attributed to antiretroviral drugs [40] provided free by the Ministry of Health. Despite such benefits, some patients still seek help from CAM. This study explores the prevalence, patterns, and perceived value of CAM among HIV patients.

## Methods

### Study design and population

This cross-sectional study was conducted among HIV clinic attendees of San Fernando General Hospital (SFGH) between November 1, 2014 and March 31, 2015. This HIV clinic is the only public clinic servicing about half (600000) of the population of Trinidad [41]. SFGH HIV clinic attendees are males (63%), Indo-Trinidadian (17.3%), Afro-Trinidadian (71.2%) and mixed descent (11.5%), and mainly in the age groups of 21–30 years (15.3%), 31–40 years (31.9%), 41–50 years (25.2%), and 51–60 years (17.3%) (data from the Monthly Report of the HIV Clinic of San Fernando General Hospital, June 2016). Inclusion criteria were age > 18 years, ability to communicate verbally, and consent to participate in the study. The desired sample size of 369 was determined to be needed in order to estimate the number of patients who used CAM (around 40%) with a margin of error of 5%. [42] Exclusion criteria were confusion (e.g. impairment of cognition and clarity) and inadequate memory recall (i.e. inability to give adequate past information) as assessed by the research assistant.

### Data collection

The data collection instrument was previously tested and used among cardiac patients in Trinidad. [17] It is a 37-item questionnaire covering demographics (seven items), current HIV condition (five items), and various aspects of CAM usage such as types of CAM, experiences, reasons, benefits, influences, effects, consequences, sources and access to CAM (25 items). Eight independent variables were considered: sex, marital status, ethnicity, educational level, employment status, religion, religiosity, and area of residence. Categorical data included employment status (unemployed, employed, retired, and unemployed due to sickness/disability) and monthly income (≤TT$2500, TT$2501–5000, TT$5001–10,000, and >TT$10000). The type of CAM used was selected from a list of different types from the NCCAM, with each expanded to the specifics developed from common practices. The list of the various CAM types comprised herbal therapy (*Aloe vera*, *Ginkgo*, ginger, turmeric, etc.), spiritual therapy (faith healing, meditation, hypnotherapy, psychic therapy, etc.), alternative systems (Chinese medicine, Indian/Ayurveda medicine, acupuncture, homeopathy), physical therapy (chiropractice, osteopathy/bone setters, massage, manual healing), energy therapies (bio-electrics magnetics, oxygen/ozone treatment), and other therapies (bloodletting cupping, ritual sacrifice, urine therapy, folk magic/sorcery (*obeah*), etc.). Each question about CAM was phrased such that the patients could respond with a list of possibilities and include others that were not listed by the NCCAM. Patients were free to choose more than one option. Face-to-face interviews were conducted with consenting patients in a private consultation room by a pre-medical student.

### Statistical analysis

Descriptive methods included frequency distribution tables and graphs. Inferential methods included tests of equality of proportions, chi-squared tests of association (e.g. Fisher’s exact test and McNemar’s test of paired proportions) between selected socio-demographic characteristics or other attribute variables and CAM use. Binary logistic regression was used to identify predictors of CAM use. All hypotheses were tested at the 5% level of significance.

## Results

A total of 343 HIV patients (response rate: 93%) participated in the study. Four patients refused to participate without reason, and 23 patients decided not to participate for various reasons, including privacy and uncomfortable feeling. Study participants were mainly females (*n* = 185, 53.9%), aged 21–40 years (*n* = 198, 57.7%), single (*n* = 197, 57.4%), Afro-Trinidadian (*n* = 178, 51.9%), Christian (*n* = 206, 60.0%), attended up to secondary school (*n* = 252, 73.5%), and were employed during the data collection period (*n* = 238, 69.4%). Almost half of the participants (*n* = 171, 49.8%) reported having a monthly income of TT$5000 or less (Table 1).

**Table 1:** Socio-demographic characteristics of the study participants

Characteristic	Number	Percent
Gender		
Male	158	46.1
Female	185	53.9
Age (years)		
< 20	26	7.6
21–30	95	27.7
31–40	103	30.0
41–50	90	26.2
51–60	3	0.9
> 60	26	7.6
Marital status		
Single	197	57.4
Married	59	17.2
Widowed	20	5.8
Divorced	24	7.0
Common law	38	11.1
Ethnicity		
Afro-Trinidadian	178	51.9
Indo-Trinidadian	79	23.0
Other (including mixed)	86	25.1
Highest level of education		
Up to primary	50	14.6
Secondary	252	73.5
Tertiary	39	11.4
Employment status		
Employed	238	69.4
Unemployed	105	30.6
Religion		
Hindu	53	29.7
Islamic	17	7.9
Christian	206	60.0
Other	39	11.3
No response	27	7.9

The internal reliability (Cronbach’s alpha) was 0.981 based on non-socioeconomic factors such as attitude, practices, and knowledge. A total of 113 (32.9%) patients answered ‘*Yes*’ to the question ‘*Have you used complementary and alternative medicine?*’, with the remaining 230 (67.1%) answering ‘*No*’. A comparison of selected demographic characteristics between CAM users and non-users is shown in Table 2.The two groups had similar profiles except for ethnicity and education. The percentage of users who were Indo-Trinidadians was more than double than the percentage of non-users who were Indo-Trinbagonians (59.8% vs. 26.5%; *p* = 0.030). The percentage of non-users who completed only up to primary school was more than double than that of users (17.7% vs. 8.0%; *p* = 0.015). The opposite was true for users and non-users who had completed their tertiary education (18.6% vs. 7.8%; *p* = 0.006).

**Table 2:** Socio-demographic characteristics of CAM users and non-users

Characteristic	CAM users	CAM non-users	*p*
Gender			
Male	47 (41.6)	111 (48.3)	0.252
Female	66 (58.4)	119 (51.7)	0.252
Age (years)			
< 20	5 (4.4)	21 (9.1)	0.135
21–30	34 (30.1)	61 (26.5)	0.522
31–40	40 (35.4)	63 (27.4)	0.134
41–50	30 (26.5)	60 (26.1)	0.927
51–60	0 (0.0)	3 (1.3)	0.544
> 60	4 (3.5)	22 (9.6)	0.052
Marital status			
Single	57(50.4)	140 (60.9)	0.081
Married	20(17.7)	39 (17.0)	0.880
Widowed	10 (8.8)	10 (4.3)	0.139
Divorced	7 (6.2)	17 (7.4)	0.823
Common law	18 (15.9)	20 (8.7)	0.066
Ethnicity			
Afro-Trinidadian	62 (54.9)	116 (50.4)	0.491
Indo-Trinidadian	18 (59.8)	61 (26.5)	0.030
Other	33 (29.2)	53 (23.0)	0.234
Employment status			
Unemployed	83 (73.5)	155 (67.4)	0.265
Employed	30 (26.5)	75 (32.6)	0.265
Education level			
Primary school	9 (8.0)	41 (17.8)	0.015
Secondary school	82 (72.6)	170 (73.9)	0.509
Tertiary	21 (18.6)	18 (7.8)	0.006

*CAM* complementary and alternative medicine

Data are the mean number (percentage)

Of the seven demographic variables measured, middle age 31–40 (odds ratio [OR] 0.287; *p* = 0.031; 95% confidence interval [CI] 0.092–0.894), 41–50 (odds ratio [OR] 0.252; *p* = 0.017; 95% confidence interval [CI] 0.082–0.778), married (odds ratio [OR] 3.048; *p* = 0.036; 95% confidence interval [CI] 1.078–8.819), with married individuals more likely to be associated with CAM use (reference group: single), and secondary school education (OR 19.599; *p* = 0.014; 95% CI 1.836–209.248) (reference group: primary school) were the only useful predictors of the likelihood of using CAM among HIV patients (Table 3).

**Table 3:** Binary Logistic Regression for CAM use among patients

Variable	OR	*p*	95% CI	
			Lower	Upper
Age				
Under 20	1			
21–30	0.672	0.588	0.160	2.828
31–40	0.287	0.031	0.092	0.894
41–50	0.252	0.017	0.082	0.778
Over 50	0.320	0.051	0.102	1.003
Sex				
Male	1			
Female	1.667	0.141	0.842	3.340
Marital status				
Single	1			
Married	3.048	0.036	1.078	8.819
Widowed	2.622	0.114	0.793	8.669
Divorced/Separated	1.576	0.589	0.303	8.186
Common Law	2.715	0.215	0.560	13.157
Ethnicity				
Afro-Trinidadian	1			
Indo-Trinidadian	0.865	0.708	0.404	1.85
Mixed	3.913	0.062	0.933	16.412
Education				
Primary school	1			
Secondary school	19.599	0.014	1.836	209.248
Tertiary	1.870	0.216	0.694	5.041
Income ($TT)				
< $2500	1			
$2501 - $5000	1.536	0.463	0.488	4.836
Over $5000	1.503	0.320	0.674	3.351
Religion				
Islam	1			
Hindu	0.643	0.566	0.142	2.904
Baptist	0.537	0.473	0.099	2.93
Anglican	2.797	0.071	0.914	8.554
Roman Catholic	2.17	0.187	0.687	6.852
Other	1.33	0.636	0.409	4.329

**Fig. 1:**
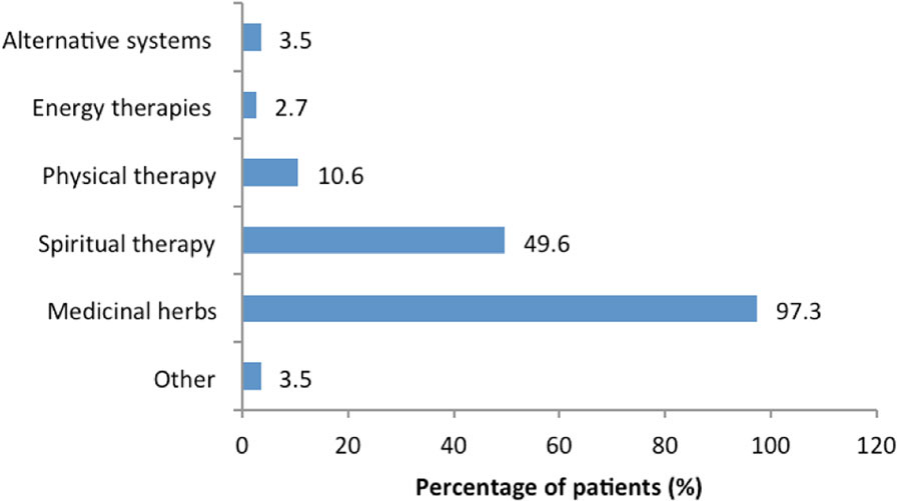
Types of CAM therapy used by HIV patients (*n* = 130)

Medicinal herbs were the most common type of CAM used (*n* = 110, 97.3%), followed by spiritual therapy (*n* = 56, 49.6%) (Fig. 1). *Aloe barbadensis Miller* (*Aloe vera*) (*n* = 54, 49.1%) was the most commonly used medicinal herb, followed by *Zingiber officinale* (ginger) (*n* = 33,29.2%), and *Allium sativum* (garlic) (*n* = 23, 20.4%) (Table 4). Only 2 patients (1.8%) used medicinal teas. Complex B vitamins were the most commonly used supplement (*n* = 69, 61.1%), followed by vitamin A (*n* = 58, 51.3%), vitamin E (*n* = 51, 45.1%), and vitamin D (*n* = 42, 37.2%). Almost half of CAM users (*n* = 56, 49.6%) resorted to spiritual therapy, all of whom sought faith healing/prayer (*n* = 56, 100%) and 13 (23.2%) practised meditation. Two (1.8%) CAM users used Chinese medicine; none used Indian/Ayurveda medicine; 1 (0.9%) used acupuncture; and none used homeopathy. Ten (8.8%) CAM users reported that they had abandoned CM for CAM in the past (1 before CM was completed, 5 after CM was completed, and 4 while CM was being used). When asked how frequently they substituted their CM treatment, 100 (88.5%) patients said they never do (i.e. they combined CAM with CM), while the remaining 13 responded ‘*often/sometimes/occasionally*’ in the ratio 2/5/6.

**Table 4:** Frequency of medicinal herb/supplement intake among HIV patients who use CAM

	Used in the past	Presently being used	Will use in the future
*Aloe vera* (*Aloe barbadensis* Miller)	54 (47.8)	51 (45.1)	54 (47.8)
Calcium	34 (30.1)	35 (31.0)	33 (29.2)
Chinese herbal medicines	7 (6.2)	7 (6.2)	8 (7.1)
Evening primrose	8 (7.1)	8 (7.1)	9 (8.0)
Flaxseed	14 (12.4)	14 (12.4)	15 (13.3)
Folic acid	37 (40.1)	32 (28.3)	33 (29.2)
Ginger (*Zingiber officinale*)	33 (29.2)	32 (28.3)	33 (29.2)
*Gingko biloba*	40 (35.4)	40 (35.4)	42 (37.2)
Ginseng	25 (22.1)	25 (22.1)	26 (23.0)
Potassium	8 (7.1)	8 (7.1)	8 (7.1)
Turmeric	8 (7.1)	8 (7.1)	7 (6.2)
Vitamin B complex	69 (61.1)	70 (61.9)	70 (61.9)
Vitamin A	57 (50.4)	57 (50.4)	58 (51.3)
Vitamin D	42 (37.2)	42 (37.2)	43 (38.1)
Vitamin E	51 (45.1)	51 (45.1)	51 (45.1)
Zinc	17 (15.0)	17 (15.0)	18 (15.9)
Omega 3	20 (17.7)	23 (20.4)	22 (19.5)
Garlic (*Allium sativum*)	23 (20.4)	23 (20.4)	23 (20.4)
Coenzyme Q10	3 (2.7)	3 (2.7)	3 (2.7)
Medicinal tea	2 (1.8)	2 (1.8)	2 (1.8)
Sure Cure products	14 (12.4)	13 (11.5)	13(11.5)
Omega XL	11 (9.7)	11 (9.7)	11 (9.7)
Special diet/supplements	5 (4.4)	5 (4.4)	5 (4.4)

*CAM* complementary and alternative medicine, *HIV* human immunodeficiency virus

Special diet/supplements: concoction of different products and/or food items to treat patient illness or to maintain wellness

Data are the mean number (percentage)

Only 17 (15.0%) patients claimed to have received any specific benefit from CAM. However, when asked to list at least one of these benefits, 13 of the 17 patients did not respond; 2 said they experienced increased energy, 1 said it assisted with sleep, and 1 said weight gain. Several patients gave reasons why they decided to use CAM (Table 5). The majority (*n* = 21, 18.6%) of CAM users said they decided to use CAM because of their desire to try anything they thought would help improve their condition. Patients stated four specific benefits which they hoped to derive from CAM use: direct treatment of their condition (*n* = 74, 65.5%), enabling them to sleep (*n* = 7, 6.2%), relieve the symptoms/side effects associated with the use of CM (*n* = 5, 4.4%), and improvement of psychological well-being (*n* = 4, 3.5%). Sixteen (14.2%) patients did not respond. The majority (*n* = 86, 76.1%) of CAM users were satisfied and 5 (4.4%) were very satisfied.

**Table 5:** Reasons for deciding to use CAM

Reasons	Number	Percent
The patient was disappointed that conventional treatment was not working	7	6.2
Conventional treatment was too toxic or damaging	1	0.9
CAM was more in keeping with personal beliefs and inner self	7	6.2
The patient felt the desire to take control of treatment	10	8.8
Conventional treatment was too mechanistic/technological and lacked human touch	0	0.0
The patient was just trying everything that could help	21	18.6
Conventional treatment was too expensive	2	1.3

*CAM* complementary and alternative medicine

**Fig. 2:**
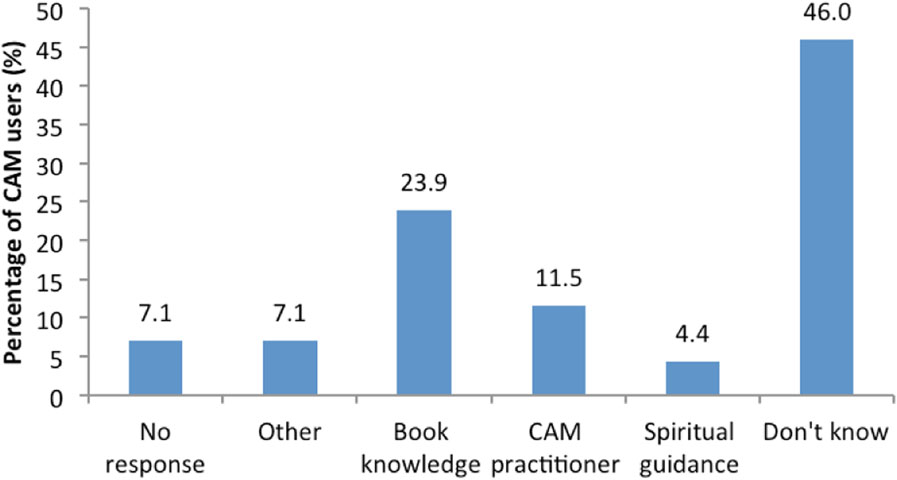
Basis for choosing the right CAM therapy

**Fig. 3:**
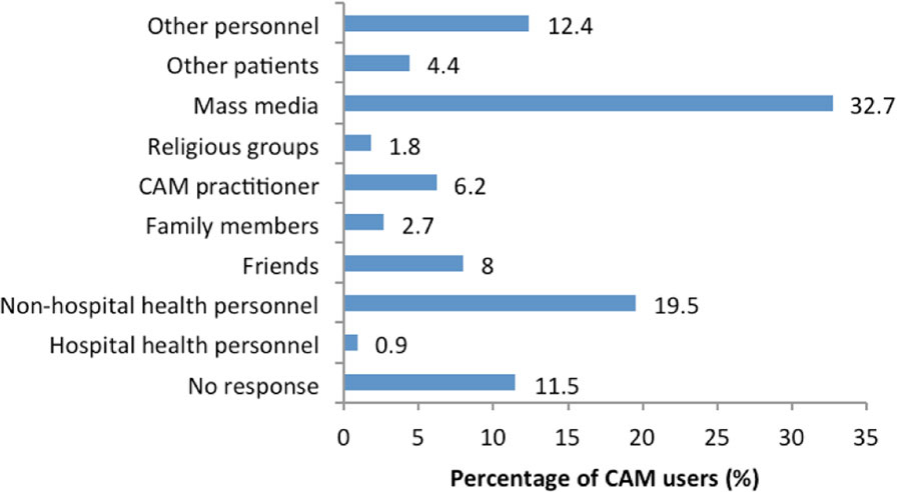
Source of awareness about the type of CAM therapy used

Fifty-two (46%) CAM users said that they did not know how to determine whether the type of CAM they were using was the right one. Others said the basis for using the right CAM resulted from book knowledge (*n* = 27, 23.9%) or their CAM practitioner (*n* = 13, 11.5%) (Fig. 2). Only 12 (10.6%) users said that their use of CAM was supervised/guided by a healthcare provider (allopathic or CAM practitioner). When asked if they thought that the more knowledgeable a person is regarding CAM, the more likely he/she is to use it, 89 (78.8%) agreed, 7 (6.2%) agreed strongly, and only 9 (5.3%) disagreed. The majority (*n* = 32, 32.7%) learned about the type of CAM they were using from the mass media; and more users were informed by non-hospital health personnel than by hospital health personnel (19.5% vs. 0.9%; *p* ≤ 0.001) (Fig. 3).

Eight (7.1%) CAM users said that they had seen an alternative medical practitioner prior to going to their medical doctor; 7 (6.2%) mentioned to the doctor that they had used/were using CAM; and none of those who did not mention their use of CAM to their doctor stated why they were reluctant to do so. Only 11(9.7%) users expressed disappointment with CAM usage: 6(5.3%) reported unwanted effects, namely acne (*n* = 1), redness (*n* = 1) and swelling (*n* = 1), and 3 were non-responses.

## Discussion

In this study, the prevalence of CAM among HIV patients was 32.9%. This is similar to the prevalence of 33.7% among HIV patients in Uganda [43] but lower than that among HIV patients in Thailand (95%) [44]. The more severe the chronic disease, the greater the likelihood of using CAM [45]. Terminal diseases are also associated with higher uses of CAM (a prevalence of 47.9% was reported in a study of cancer patients in Mongolia [46]). In this study, however, the prevalence of CAM among HIV patients was relatively lower. The relatively low prevalence of CAM use in Trinidad may also result from fear of using CAM along with antiretroviral drugs that have proven effective, and the lack of experience with CAM for HIV infection.

The use of CAM was more common among females (58.4%) and young (21–40 years) patients (61.9%). This is in keeping with a review by Lorenc and Robinson, who found younger females were more likely to use CAM [47]. In our study, it was found that income was not associated with use of CAM as was found by Idung and Abasiubong [48]. In another study, female gender and higher income (greater than US$250000 per year and higher literacy) [49] were associated with higher CAM usage. Education was also positively associated with CAM use [50]. This study reports higher use of CAM with secondary education compared with Primary school education.

CAM was more common among Indo-Trinidadians (59.8%). Another study conducted among Africans (51.7%), Caucasians (31.1%), and Hispanics (13.7%) reported that the majority of CAM users were of African descent [51]. Predictors of CAM among HIV patients revealed that the only predictors of CAM use among HIV patients were middle age, married and secondary school educated.

The majority of CAM users (97.3%) used herbal medicines, the commonest being *Aloe vera* (49.1%). This is of concern since many herbs are known for their toxic effects. *Ginkgo biloba* extract used by some patients has led to a virological breakthrough [52]. Though *Aloe vera* is fairly safe, there is still the possibility of drug interactions, including with antiretroviral drugs [53]. Spiritual therapy was also widely practiced, which is in agreement with the work by Cotton et al. [54] The majority of CAM users used at least one herb.

HIV drugs may also lead to side effects such as diarrhoea, nausea and vomiting, rashes, lipodystrophy, and increased risk of heart attack. Antiretroviral drugs such as azidothymidine (AZT) may cause headaches and fatigue, stavudine (d4T) may cause peripheral neuropathy, protease inhibitors (PIs) may cause retinoid toxicity, and non-nucleoside reverse transcriptase inhibitors (NNRTIs) may cause hypersensitivity reactions [55].

The desire of patients to use CAM was attributed to their willingness to try anything. They hoped to treat the disease directly, improve sleep, relieve the symptoms/side effects associated with the use of CM, and improve psychological well-being. CAM usage is attributed to the increasing demand and expectations for more holistic and comprehensive care [6]. As previously reported by similar studies, people were discouraged by the inadequacy of CM, mechanistic and lack of human touch of CM, costly CM, toxic CM, and ineffective CM (push factors); and were encouraged by the synchrony with patients beliefs, the ability to take greater control of one’s life and other CAM (pull factors). “Push” factors encompassed dissatisfaction with conventional treatments, whereas “pull” factors included a desire for more holistic and “natural” approaches, and a greater philosophical congruence with CAM [56]. CAM is regarded as being “natural” and “safe” [57], effective [58], and effective to reduce adverse effects [7]. CAM users also appreciate the feeling of control, coping, and adjustment [59].

The greatest influence for CAM usage came from the mass media (32.7%), followed by non-hospital health personnel (19.5%). Family members accounted for 2.7% only. This may reflect the limited extent of family and social support for these patients who end up relying on the mass media. A study in Bangkok on HIV/AIDS patients showed that the influence of friends (50.22%), family (45.33%), and health service providers (44.44%) was much higher than in this study [59]. CAM usage also depends on the social, cultural, economic, and traditional influences [60]. Although there is the potential for major negative consequences, the majority of CAM users (76.1%) were satisfied and a few (4.4%) were very satisfied. This is in agreement with the high levels of satisfaction (69.2%) found among HIV patients in a small cross-sectional study in India [61]. The majority of CAM users (88.5%) refused to substitute CM by CAM. The failure to abandon CM reflects the continued value of CM and the desire to try something different to add value to their outcome.

Many patients (46%) did not know if the type of CAM they were using was the right one although they became aware from sources such as books and their CAM practitioner. Furthermore, only 10.6% of CAM users were supervised/guided by a healthcare provider/professional. In fact, according to Wahner-Roedler et al., few doctors (24%) are prepared to refer patients to a CAM practitioner [62]. In addition, doctors are unprepared to communicate and treat with CAM issues [63]. In this study, few healthcare providers (6.2%) were informed about previous or present CAM usage. Another study reported that 76% were poorly informed about herbal medicines and 45.6% had “very poor” or “quite poor” knowledge of CAM. [64] Liu et al. [65] found that 36% of patients disclosed CAM practices to their physician. Furthermore, more than 77% of physicians do not discuss CAM treatments with their patients [66]. This may not be in the best interest of patient care since vital information required for the management of patients is lost. Nonetheless, even with the high non-disclosure rate found in this study, its use continues unregulated as a result of the lack of regulations in Trinidad and Tobago [67].

Low reporting to healthcare providers, high satisfaction rating with CAM usage and poor monitoring makes CAM a major public health problem. There are numerous herb–drug interactions [68] and lack of evidence in many cases to support their claims [8]. Major health consequences can result, including delayed treatment, disease complications, and occasionally death. CAM therapies are also of questionable safety and efficacy [69]. Public safety and a scientific basis for CAM usage have been emphasised by Trinidad and Tobago’s Chief Medical Officer [70] and the World Health Organization (WHO) [71]. The simultaneous practice of CAM and CM needs greater understanding, communication, and integration of both. Policy makers, implementers, and customers need greater attention on CAM usage.

The limitations of this study include the selection of patients from a single HIV public clinic located at the SFGH in South Trinidad. The attendees of the clinic tended to be of a lower socioeconomic status and educational level, which is not fully representative of the population. There may be recall bias. To assist patient recall and to maintain uniformity on the meaning of CAM, patients were provided with a list of most common types of CAM and a list of options to choose from in various questions about CAM. The negative stigma attached to HIV patients is still felt by some and they may be reluctant to discuss their condition freely. To minimise negative feelings, patients were encouraged by the nurse and interviewed in private rooms in the clinic.

## Conclusions

The prevalence of CAM use was relatively high (32.9%) among HIV patients. The most used types of CAM were herbal therapy (97.3%) and spiritual therapy (49.6%). CAM usage was more common among young and female patients. Being of middle age, married, and secondary school educated were the only useful predictors of CAM use. Its use was driven by cost, individual beliefs, willingness to try anything, and perceived benefits such as wellbeing, relaxation, and counteracting the side effects of CM. The majority (over 90%) of CAM users were satisfied with CAM. However, the majority are unwilling to disclose such information to their CM practitioners. HIV patients receive information mainly from the mass media and non-hospital health personnel. Non-disclosure, high satisfaction levels, and the simultaneous use of both CM and CAM make CAM practice a major public health problem because of the possibility of delayed treatment as well as herb–herb and herb–drug toxicity. Patients, the community, and CM and CAM practitioners need to have adequate information to guide patients in effective and appropriate usage, to minimise the risks of CAM, and to give greater empowerment to CAM users. This will also assist in providing safe and effective health care.

## Abbreviations

AZT: Azidothymidine
CAM: Complementary and alternative medicine
CM: Conventional medicine
CMO: Chief medical officer
d4T: Stavudine
HCP: Healthcare provider
HIV: Human immunodeficiency virus
NNRTI: Non-nucleoside reverse transcriptase inhibitor
PI: Protease inhibitor
SFGH: San Fernando General Hospital
WHO: World Health Organization
